# ICP Trace Element Analyses from Fusion Dissolution

**DOI:** 10.6028/jres.093.117

**Published:** 1988-06-01

**Authors:** D. Brown, G. Legere, P. Burgener

**Affiliations:** Technical Services Laboratories, 1301 Fewster Drive, Mississauga, Ontario, Canada L4W 1A2

## Lithium Carbonate-Boric Acid Fusion Digestion for ICP

The fusion digestion procedure of using a mixture of lithium carbonate and boric acid to prepare silicate rocks for analysis is not new. At Technical Service Laboratories (TSL) this fusion procedure has been used to perform “whole rock” analysis by ICP since 1979. Others, [1–4], have published values for the major rock constituents by ICP, X-ray, and other analytical techniques after a lithium carbonate-boric acid fusion. In the last 3 years at TSL however, minor elements were also determined on the same solution from which the majors were measured. With careful quality control procedures and a constantly updated interference correction matrix, minor elements can be also obtained with detection limits as low as 1 ppm for some elements. The unique buffering effect of the fluxing material produces stable background levels which allows for easier correction of interelement concomitants.

As part of our ICP quality control program, major and minor element data have been compiled on international rock standards. The accuracy of the minor element data produced over the 3-year time period demonstrates the ability of the fusion to produce detection limits similar to that of other dissolution methods used prior to analysis by ICP.

## Procedure

The digestion procedure used is similar to other digestion procedures using lithium metaborate published by Walsh [2], although several changes have been developed to make routine analysis simpler.

Approximately 200 mg of previously dried and sieved (−200 mesh) sample is weighed accurately to ±0.1 mg and is added to 1.50 g of dried fluxing material in a graphite crucible. The fluxing material is obtained by combining ground 99.9% pure boric acid and 99.99% pure lithium carbonate in a ratio of 2:1 by weight. The flux mixture is thoroughly blended, dried overnight and weighed into a prepared crucible. The flux-sample mixture is preheated to 400 °C for 1/2 hour and then heated to almost 1200 °C in a nitrogen atmosphere. The hot melt is gently swirled in the crucible and quickly poured into a 250 mL polypropylene bottle containing a mixture of 50.00 g of 16% v/v reagent grade HNO_3_ and the Cd internal standard. The bottle is then placed on a rotary shaking tray for at least 2 hours, After shaking, the solutions are diluted with 70.00 g of double deionized water and decanted into test tubes ready to be run on the ICP. By this method, one person can completely prepare 120 samples in an 8-hour period.

For analysis, the samples are placed on a Gilson linear belt auto-sampler with a 30-second rinse cycle and a 30-second analysis time. A summary of the instrumentation and operating parameters used for the analyses are summarized in tables 1 and 2.

Calibration is achieved for the major oxides and Ba, Sr, Zr by running five international rock standards MRG-1, SY-2, NIM-G, NIM-L, and NBS Dolomite with a reagent blank and deriving an intensity vs concentration curve from these standards. For the minor elements, a synthetic solution is created by adding 1 or 2 mL of 1000 mg/L Spex or Inorganic Ventures plasma grade standards to a reagent blank and diluting as normal. This solution gives 5000 and 10000 ppm equivalence in the rock. With the advantage of the linear intensity vs concentration of the ICP source, and the buffering effect of the flux material, one point calibration has proven sufficient for the levels of trace elements one finds in silicate rocks. By having a fusion flux to buffer the solution, spectral baselines are more stable from sample to sample, making it easier to perform spectral overlap corrections from either a direct line or a wing.

Interelement interference correction is achieved by running blank reagent solutions doped with typical levels of the interfering elements found in whole rock solutions. Spex plasma grade standards were again used for the doping material where possible; otherwise, solid pure oxide forms of the element were obtained from Johnson Matthey and prepared under the same conditions as the samples.

The intensity obtained in an element channel is stored in the form of a matrix for each of the interfering elements. This matrix is updated monthly or when any of the operating parameters are changed. The matrix, after reagent blank subtraction, is used to correct for the interfering element in each analyte channel.

## Results and Discussion

Analyses of selected international standards are given in table 3. Additional data are available for other standards including NIM-N; JR-1, JA-1, and JB-2 (Japan); NBS 1633a, and NBS 98a. As is usual, there are several precautions to note for this fusion using graphite crucibles. As indicated by Ingamells [1] some of the flux-sample mixture may stick to the walls of the crucible after the hot melt has been poured into the polypropylene bottle. To collect the remainder from the crucible, it is allowed to cool and the walls are gently scraped with a spatula and reheated to almost 1200 °C. The remainder is poured into the already swirling solution, and the stirring action is resumed. By doing this, the crucible will be completely free of sample and flux.

To minimize the sticking of the flux mixture to the walls of the crucible, the walls are gently scraped with a reaming tool to build up a carbon layer on the surface. Nevertheless, it has been found that no amount of pretreatment can eliminate entirely the sticking of the flux to walls of the crucibles for every sample.

The accuracy of this method of analysis for the major oxides is predominantly dependent upon two factors; the accuracy of the standards used to form the calibration curves and the stability of the plasma emission source. The latter of these problems covers the fluctuations of viewing height, power supplied by the radio frequency generator, and analyte transportation to the plasma. In this case, power to the plasma is monitored by an auto-controller. But no attempt has been made to monitor the effects of any power fluctuations at the plasma itself (i.e., monitoring an argon line). Any short term fluctuations in power are smoothed by the length of the 30-second integration time.

The analyte transport to the plasma is monitored via the Cd internal standard. The sole purpose of the internal standard is to account for variations in nebulizer efficiency from sample to sample. One internal standard cannot, alone, accurately monitor changes in power levels and sample dilution errors (even though it appears as if it does when plasma emission conditions remain stable). In our experience, however, no internal standardization of any form can account for changes in viewing height. The gas flows through the nebulizer orifice must remain very stable to keep the analytical zone constant for extended periods of time. If this condition is not met, gross changes in calibration and interelement spectral interferences will occur.

The major source of analytical error for the minor elements is predominantly determined by how accurately the emission line profiles can be corrected for interelement interferences. It is commonly the case where one finds that what one assumed to be a direct line overlap from a concomitant is simply contamination in the spectral correction solution. Yet the biggest sources of error when performing these spectral corrections routinely are the stability of the position of the observation zone in the plasma, and the temperature variations of the spectrometer itself. Unless the spectrometer has an independent temperature control, the room in which it is situated should have its temperature and humidity stabilized to within a few degrees. Otherwise, any matrix corrections performed must take into account drift of the position of the analytical lines with respect to the nearby interfering elements.

A change of observation height within the plasma can lead to incorrect matrix corrections since emission line strengths of different atomic and ionic populations vary with viewing height. Either the nebulizer’s characteristics (e.g., gas flow) must remain constant, or, careful monitoring and adjustment must be done continuously with an apparatus such as a mass flow controller.

For our purpose, the use of the Legere Teflon nebulizer has eliminated gas flow variations in the nebulizer itself, thus ensuring a constant observation zone within the plasma for weeks at a time without need of adjustment.

A safe detection limit of 5 ppm is found for the trace elements (Ba, Co, Cr, Cu, Sc, Sr, Y, Zn, and Zr) in silicate rocks fused with lithium carbonate and boric acid, except for Be, which has an extremely sensitive line, and has a detection limit of 1.

However, in table 3, only values catalogued over the last 3 years are given. The additional elements included in the list above are those which have only recently been measured. It is hoped that in future months detection limits of 5 ppm or better can be achieved for Th, W, Sb and Sn,

## Figures and Tables

**Table 1:** Summary of instrumentation and operating parameters used for TSL analyses

Spectrometer #1	Jarell-Ash 975 Atomcomp, 0.75 m, 2400 grooves/mm Fixed 50 µm slits, with PDP-8 and IBM pc computers
Spectrometer #2	Jarrell-Ash 975 Atomcomp, 0.75 m, 2400 groves/mm Scanning spectrum shifter of 50 µm slits with IBM pc computers
Plasma torch	Mak torch to fit 8″ Jarrell-Ash from Sherritt Gordon Research
Argon flows	Outer 10 L/min Support 1.5 L/min Nebulizer 0.8 L/min
Generator	Plasma Therm Model HFP-2000D, 27.1 MHz, with Autopower control
RF Power	Presently operating at 0.75 kW but in past have operated at 1 kW incident power
Nebulizer	Legere high salt nebulizer
Load coil	Presently using 1 1/2 turn copper oil but have previously used 3 1/2 turn copper coil

**Table 2:** Analytical wavelengths (Å) used for ICP analyses

Spectrometer #1									
Si	I	2881.6	Ti	II	3349.4	Cu	I		3247.5
AI	II	3961.5	Mn	II	2576.1	Ni	II	2x	2316.2
Fe	II	2599.4	P	I	2136.2	Sr	II		4077.7
Ca	I	4226.7	Ba	II	4554.0	V	II		2924.0
Mg	II	2795.5	Be	I	2348.1	Zn	II	2x	2062.0
Na	I	5889.9	Co	II	2286.0	Zr	II		3438.2
K	I	7664.9	Cr	II	2677.2				
Spectrometer #2									
Th		2837.3	Cu	I	3247.5	Co	II		2286.0
Y	II	3710.3	V	II	2924.0	Zn	I		2138.6

**Table 3:** TSL ICAP analyses on selected international rock standards

GA (Fr)				MICA-Fe (Fr)			NIM-P			NBS 70a		
	TSL values Mar 84–Jul 85 (*N* = 17)		Abbey [5]	TSL values Jul 84–Aug 86 (*N* = 16)		Abbey [5]	TSL values Aug 84–Apr 86 (*N* = 19)		Abbey [5]	TSL values Sep 84–Apr 86 (*N* = 16)		NBS values
Average *s*^a^				Average *s*			Average *s*			Average *s*		
SiO_2_	69.62	0.35	69.96	34.34	0.24	34.55	51.21	0.62	51.10	67.27	0.38	67.10
Al_2_O_3_	14.68	0.10	14.51	19.51	0.23	19.58	4.17	0.06	4.18	17.97	0.14	17.90
Fe_2_O_3_	2.75	0.02	2.77	25.44	0.25	25.76	12.67	0.16	12.76	0.07	0.02	0.07
CaO	2.47	0.02	2.45	0.48	0.14	0.43	2.61	0.07	2.66	0.11	0.02	0.11
MgO	0.93	0.01	0.95	4.60	0.04	4.57	25.30	0.46	25.33	0.03	0.01	
NaO	3.47	0.06	3.55	0.20	0.02	0.30	0.26	0.04	0.37	2.42	0.07	2.50
K_2_O	4.01	0.11	4.03	8.83	0.15	8.79	0.10	0.05	0.09	11.75	0.22	11.80
TiO_2_	0.37	0.01	0.38	2.59	0.02	2.51	0.19	0.01	0.20	0.00	0.00	0.01
MnO	0.09	0.00	0.09	0.35	0.00	0.35	0.22	0.00	0.22	0.00	0.00	
P_2_O_5_	0.12	0.01	0.12	0.40	0.02	0.45	0.04	0.02	0.02	0.01	0.01	
BaO	0.09	0.00	0.09	0.02	0.00	0.02	0.00	0.00	0.01	0.01	0.00	0.02
SrO	0.04	0.00	0.04	0.00	0.00	0.00	0.00	0.00	0.00	0.01	0.00	
ZrO	0.02	0.00	0.02	0.11	0.00	0.11	0.00	0.00	0.00	0.00	0.00	
Average *s*				Average *s*			Average *s*			Average *s*		
Be	3	1	4	7	1	8?	<1	1	<1	1	1	
Co	8	5	5	25	5	20	110	5	110	5	5	
Cr	10	5	12	90	5	90	2.1%	0.3%	2.4%	<5	5	
Cu	18	10	16	35	10	4?	10	5	18	5	5	
V	37	5	38	135	10	135	250	5	230	<5	5	
Zn	64	5	80	1210	30	1300	120	10	100	<5	5	

a *s* = standard deviation.
